# Total hip arthroplasty has higher complication rates in stiff spine patients: a systematic review and network meta-analysis

**DOI:** 10.1186/s13018-022-03237-8

**Published:** 2022-07-16

**Authors:** Sung Huang Laurent Tsai, Ngi Chiong Lau, Wei Cheng Chen, Ruei-Shyuan Chien, Eric H. Tischler, Tsai-Sheng Fu, Dave Wei-Chih Chen

**Affiliations:** 1 Department of Orthopaedic Surgery, Chang Gung Memorial Hospital, Keelung Branch, Keelung, 204 Taiwan; 2 School of Medicine, Chang Gung University, Taoyuan, 333 Taiwan; 3 Department of Orthopaedic Surgery, Chang Gung Memorial Hospital, Linkou Branch, Linkou, Taiwan; 4 Johns Hopkins Bloomberg School of Public Health, Baltimore, MD USA; 5 Department of Orthopaedic Surgery and Rehabilitation Medicine, State University of New York, Downstate Medical Center, Brooklyn, NY USA

**Keywords:** Total hip arthroplasty, Ankylosing spondylitis, Spinal fusion, Outcomes

## Abstract

**Background:**

Ankylosing spondylitis (AS) and spinal fusion (SF) classified as stiff spines have been associated with the increased rate of complications following total hip arthroplasty (THA). However, the differences between the two cohorts have inconsistent evidence.

**Methods:**

We searched for studies comparing complications among stiff spine patients, including SF and AS, who underwent THA in PubMed/MEDLINE, Embase, Cochrane CENTRAL, Web of Science, and Scopus until March 2021. Studies detailing rates of mechanical complications, aseptic loosening, dislocation, infection, and revisions were included. We performed network meta-analyses using frequentist random-effects models to compare differences between cohorts. We used P-score to rank the better exposure with the lowest complications.

**Results:**

Fourteen studies were included in the final analysis. A total of 740,042 patients were included in the systematic review and network meta-analysis. Mechanical complications were highest among SF patients (OR 2.33, 95% CI 1.86, 2.92, *p* < 0.05), followed by AS patients (OR 1.18, 95% CI 0.87, 1.61, *p* = 0.82) compared to controls. Long Spinal Fusions had the highest aseptic loosening (OR 2.33, 95% CI 1.83, 2.95, *p* < 0.05), dislocations (OR 3.25, 95% CI 2.58, 4.10, *p* < 0.05), infections (OR 2.14, 95% CI 1.73, 2.65, *p* < 0.05), and revisions (OR 5.25, 95% CI 2.23, 12.32, *p* < 0.05) compared to AS and controls. Our results suggested that SF with longer constructs may be associated with higher complications in THA patients.

**Conclusions:**

THAs following SFs have higher mechanical complications, aseptic loosening, dislocations, and infections, especially with longer constructs. AS patients may have fewer complications compared to this cohort.

**Supplementary Information:**

The online version contains supplementary material available at 10.1186/s13018-022-03237-8.

## Background

The prevalence of ankylosing spondylitis (AS) is estimated to be between 0.1% and 1.4% worldwide [1, 2]. AS is a seronegative inflammatory condition that typically affects patients between 20 and 40 with presenting symptoms involving the spine, sacroiliac joints, and hip joints [3]. Hip osteoarthritis secondary to chronic synovitis has been reported between 19 and 50%, with 90% presenting with bilateral symptoms [3]. Symptoms range from synovitis, entheseal inflammation, involvement of medullary bone, and progressive degeneration secondary to osteoarthritis. These pathological skeletal changes ultimately contribute to fixed flexion contractures [4].

Total hip arthroplasty (THA) is an effective treatment for degenerative changes and pain that occur in AS patients [5, 6]. Approximately 12–25% of the AS patients will undergo a THA after 30 years of age, with most patients less than 50 years of age [7]. Although AS patients are significantly younger than osteoarthritic or avascular necrotic patients requiring THA, additional challenges of operating among AS patients exist. US Nationwide Inpatient database study reported increased risks of wound complications, mortality, postoperative cardiovascular events, and central nervous complications in AS compared to patients with OA undergoing THAs [8]. AS patients reported 2.2 and 3.6 times higher revision and dislocation rates compared to non-AS cohorts [9, 10].

Exposure of the hip joint during THA among stiff spine patients may be limited due to heterotopic ossification (HO), ankylosis, or acetabular protrusio [11]. The bone is often soft due to regional osteoporosis. Furthermore, progressive disease can develop a kyphotic spine, resulting in hip joint extension and a posteriorly rotated pelvis to compensate for sagittal imbalance to maintain a horizontal visual field. Various osteotomy techniques including pedicle subtraction osteotomy and vertebral column resection have been described to correct the sagittal deformity.

The need for accurate implanting is critical to achieving successful postoperative outcomes. Prior literature has demonstrated that spinal stiffness is associated with an increased rate of postoperative THA dislocation [7–12]. This is shown not only in AS, but also SF patients. Katakam et al. reported a total of 277 patients and demonstrated an increased dislocation rate of 11.85% dislocations from the SF group and 2.82% from the AS group after THA [13]. These findings may be attributed to spinopelvic immobility, resulting in a lower pelvic incidence, inadequate pelvic tilt while standing and sitting, and increased positive sagittal balance.

Given that the current stiff spine and THA relationship exists among a diverse demographic, this systematic review and network meta-analysis aims to advance the comprehensive surgical evaluation of the spinopelvic pathology by identifying factors associated with postoperative THA complications among stiff spine patients.

## Methods

### Research protocol and search question

The PICO search protocol framework was followed to address the hypothesis: This study aimed to answer the following the research question: Do stiff spine patients undergoing THA, compared to the non-stiff spine cohort, show higher complication rates (including mechanical complications, aseptic loosening, dislocations, infections, and revision rates)? Preferred Reporting Items for Systematic Reviews and Meta-Analyses (PRISMA) statement guidelines were utilized for study protocol review and registered in PROSPERO (CRD42020184137) [14] (Additional file 1).

### Eligibility criteria and primary outcome

Studies were eligible if they met the following criteria: (1) included adult patients who underwent total hip arthroplasty; (2) were observational studies or randomized controlled trials published in English language; and (3) reported dislocations, aseptic loosening, infection rates, periprosthetic fractures, and revision rates as primary outcomes. Mechanical complications were included if the complication was reported from the article. We defined mechanical complications as periprosthetic fractures, aseptic loosening, and dislocations according to the literature. Relevant exclusion criteria included: (1) single-arm follow-up studies, case reports, small case series of less than 10 patients, reviews, basic science experiments, and animal or cadaver studies; (2) studies including patients with severe infection or immunosuppression; and (3) conference abstracts without corresponding full-length papers.

### Search strategy and study selection

We searched PubMed/MEDLINE, Embase, Ovid, Cochrane Central Register of Controlled Trials (CENTRAL), Web of Science, and Scopus for articles in a systematic approach utilizing the combination of keyword and Medical Subject Headings (MeSH) adjusted for each database. We presented the detailed search strategy in Additional file 1. We also searched the reference lists in the included studies to obtain additional studies.

Two reviewers independently evaluated eligible studies by titles and abstracts and then reviewed the full text of relevant articles for further qualification. All disagreements between reviewers were resolved through discussion to reach a consensus, and the third reviewer was consulted if necessary.

### Data collection and quality assessment

Two independent reviewers extracted all data onto a pre-planned Microsoft Excel spreadsheet (version 16.32). Data fields included study characteristics (authors, year of publication, region of study, study design), study arms, patient age, sex, and follow-up months. The quality of included studies was also assessed independently by two independent reviewers. All included study quality metrics were graded using the Newcastle–Ottawa Scale [15]. ROBINS-1 and the Risk of Bias of the Cochrane Collaboration (ROB 2.0) were used for quality assessment [16]. All discrepancies were resolved by discussion, and a third reviewer was consulted if necessary.

### Statistical analysis and quantitative data synthesis

A network meta-analysis was performed with the use of the frequentist model from the netmeta package. Network symmetry and geometry of the evidence were evaluated with a network plot. Adjusted funnel plots were utilized to identify publication bias. Symmetry around the effect estimate line indicated the absence of publication bias. Outcomes were summarized using a random-effects model with the odds ratio (OR) effect measure for binary outcomes. Results were visualized in forest plots using control cohorts as the reference group. P-score was utilized for outcome ranking [17]. To account for study heterogeneity, DerSimonian and Laird [18] approach was implemented. Heterogeneity of the effect estimate within studies was quantified by Higgins et al. [19]. The consistency between the direct, indirect, and network evidence was summarized by node-splitting methods [20]. Network meta-analysis is an advanced form of meta-analysis in which three or more multiple comparisons are being compared with a common comparator [17]. The meta-analysis applied in the current study used a mixed comparison with generalized linear mixed models to analyze the direct and indirect comparisons among the NMA [21]. Specifically, indirect comparisons were calculated by the transitivity; thus, the differences between treatments A and B can be calculated from their comparisons with a third treatment, C. To compare multiple treatment arms, we combined direct and indirect evidence from the included studies [20]. The global statistical heterogeneity was assessed across all comparisons using the τ2 measure from the netmeta statistical package [22]. Estimates of τ2 of approximately 0.04, 0.16, and 0.36 were considered to represent a low, moderate, and high degree of heterogeneity, respectively. Statistical analysis was conducted using the R software (Version 3.6.0).

### Subgroup analysis

Subgroup analyses were performed based on treatment comparisons to evaluate within-study heterogeneity. As for studies including SF patients, outcomes were analyzed separately if comparison of short (1–2 levels) and long (3–7 levels) spinal fusion data was provided, in order to evaluate the extent of spinal fusion prior to THA.

## Results

### Literature search and selection process

**Fig. 1:**
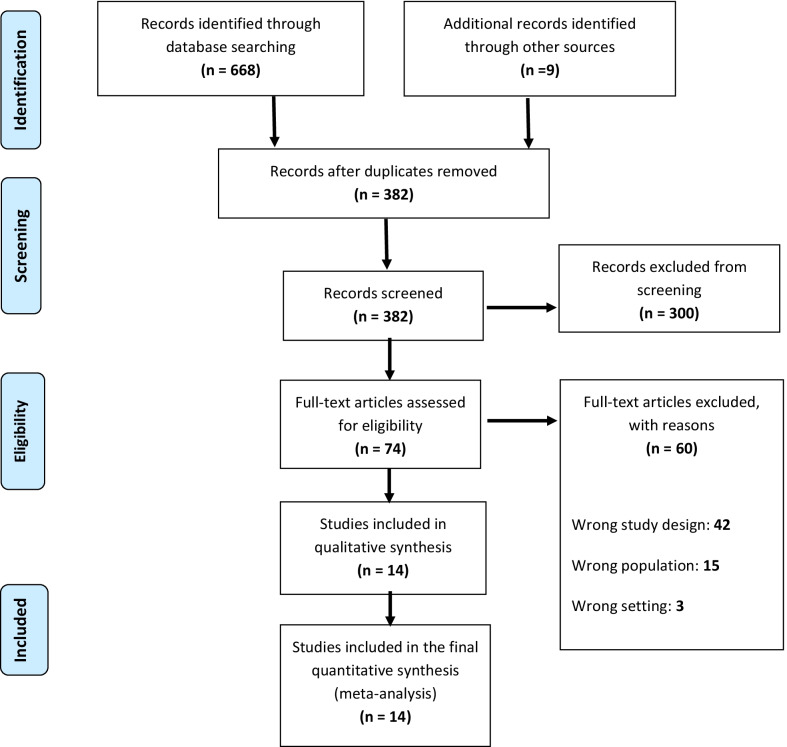
PRISMA flow diagram showing the literature review, search strategy, and selection process

A total of 668 articles were identified through database searching. Nine additional articles were identified after evaluation of references. After duplicate removal, 382 articles remained. Following screening by titles and abstracts, 74 full-text articles were reviewed for eligibility, of which 14 studies met inclusion criteria for network meta-analysis (Fig. 1).

### Study characteristics, cohort description, and treatment definition

Fourteen studies with a total of 3,418,499 patients were identified. All the studies were cohort studies with either institutional database or national registry database. A total of 740,042 stiff spine patients were included, of whom 88.1% (651,649/740,042) and 11.9% (88,393/740,042) were AS and SF patients, respectively. Study demographics were reported from the following countries: the USA (11 studies; 739,919 patients), Argentina (1 study; 9 patients), Korea (1 study; 30 patients), and Singapore (1 study; 84 patients). 651,649 patients are AS, and 88,393 patients are SF.

Of the patients included, 61.9% of participants were male. AS and SF patient mean ages were 67.0 (range 39.6–69 years) and 66.7 (range 66.3–71.6), respectively. Of the total of 88,393 SF patients, 22% (19,480/88,393) and 5.6% (4916/88,393) clearly reported short (SSF) and long (LSF) posterior spinal fusion, respectively (Table 1). The median follow-up time was 28 months.

**Table 1:** Characteristics of studies included in the network meta-analysis

Study	Design	Group	Age (years)	Sex (M/F)	Follow-up (months)	Location	ROB	Mech	Asep	Disl	Infe	Revi
Ward [23]	Retrospective	AS	61.1 ± 10.8	1924/849	12	USA	4	Y	N	N	Y	Y
Banos [24]	Retrospective	AS	48 (42–53.5)	8/1	144	Argentina	6	Y	Y	N	N	N
Blizzard [34]	Retrospective	AS	65–69	248,205/400490	24	USA	7	Y	Y	Y	Y	Y
Lee [25]	Retrospective	AS	39.6	26/4	69	Korea	8	Y	Y	N	Y	N
Katakam [13]	Retrospective	AS,SF	58.07 ± 15.6, 66.32 ± 10.4	75/67, 53/82	74	USA	8	Y	N	Y	N	N
Barry [26]	Retrospective	SF	68.5 ± 9.2	21/14	3	USA	5	Y	Y	Y	Y	Y
Bedard [27]	Retrospective	SF	71.6 (59–82)	0/328	59	USA	8	Y	N	Y	N	N
Buckland [10]	Retrospective	SSF,LSF	NA	5030/9638	12	USA	7	Y	N	Y	N	N
Loh [28]	Prospective	SF	67.59 ± 8.32	16/68	24	Singapore	7	Y	N	Y	Y	N
York [29]	Retrospective	SSF,LSF	63.5	7/24	32	USA	6	Y	N	Y	N	Y
Salib [30]	Retrospective	SF	71 ± 9	43/54	72	USA	8	Y	Y	Y	N	Y
Sing [9]	Retrospective	SSF,LSF	NA	3282/6412	24	USA	6	Y	Y	Y	Y	Y
Perfetti [31]	Retrospective	SF	64.5	343/591	12	USA	7	Y	N	Y	N	Y
Malkani [32]	Retrospective	SF	NA	NA	60	USA	7	Y	N	Y	Y	Y

Columns indicate whether a given complication from each study was included in the analysis. Follow-up period denoted refers to the mean follow-up period in months unless otherwise stated. Risk of bias was assessed using the Newcastle–Ottawa Scale except where otherwise stated. Yrs, years; M, males; F, females; mo, months; ROB, Risk of bias assessment with the Newcastle–Ottawa Scale; Mech., Mechanical Complications; Asep., Aseptic Loosening; Disl., Dislocations; Infe., Infection; Revi. Revision; Y, yes; N, no; and NA, not applicable

### Methodological quality and assessment of risk of Bias

The Newcastle–Ottawa Scale [15] was used to assess study methodological quality. Criteria include: selection of the study groups; comparability of the groups; and ascertainment of the exposure of interest for case–control or cohort studies. Scores are reported in Table 1. Additional ROBINS-I assessments for the observational studies are reported in Additional file 1.

### Mechanical complications

**Fig. 2:**
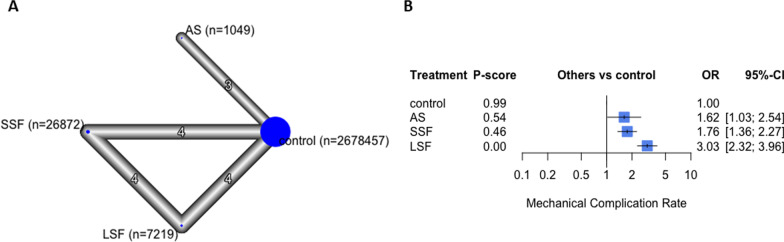
The mechanical complications analysis. In the network plot **a**, each ellipse (node) represents a specific exposure, with its size proportional to the number of cohorts in the node. Connections between nodes represent direct comparative studies between exposures, with the weight of the connecting lines representing the volume of direct comparative evidence. **b** Forest plot depicting the estimated odds ratio (OR) for each exposure pair denoted by the black square, and its associated 95% CI denoted by the width of the line, with a higher OR indicating a higher odd of complications of the group. The higher P-score demonstrates the better exposure. The 95% PI refers to the 95% prediction interval for each point estimate

Fourteen studies with 2,348,505 participants reported this outcome [4, 9, 10, 13, 23–32]. The overall structure is displayed in Fig. 2. Spinal fusion patients reported the highest odds ratio of postoperative THA mechanical complications (OR 2.33; 95% CI 1.86, 2.92, *p* < 0.05), followed by AS patients (OR 1.18, 95% CI 0.87, 1.61, *p* = 0.82), when compared to control patients (Additional file 1). Subgroup analysis showed that LSF had the highest mechanical complications (OR 3.03; 95% CI 2.32, 3.96, *p* < 0.05), followed by SSF (OR 1.76; 95% CI 1.36, 2.27, *p* < 0.05), followed by AS (OR 1.62; 95% CI 1.03, 2.54, *p* < 0.05), compared to controls (Fig. 2).

### Aseptic loosening

**Fig. 3:**
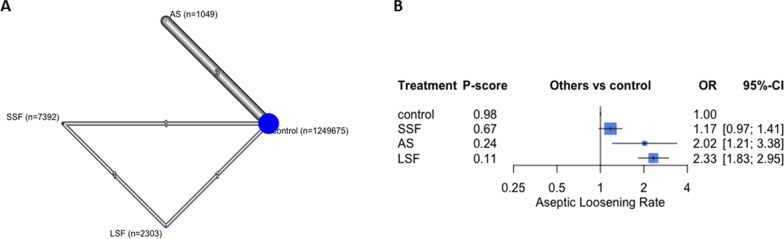
The aseptic loosening analysis. In the network plot **a**, each ellipse (node) represents a specific exposure, with its size proportional to the number of cohorts in the node. Connections between nodes represent direct comparative studies between exposures, with the weight of the connecting lines representing the volume of direct comparative evidence. **b** Forest plot depicting the estimated odds ratio (OR) for each exposure pair denoted by the black square, and its associated 95% CI denoted by the width of the line, with a higher OR indicating a higher odd of complications of the group. The higher P-score demonstrates the better exposure. The 95% PI refers to the 95% prediction interval for each point estimate

Six studies with 1,260,419 participants reported this outcome [4, 9, 24–26, 30]. The overall structure is shown in Fig. 3. Overall, LSF reported the highest aseptic loosening estimate (OR 2.33; 95% CI 1.83, 2.95, *p* < 0.05), followed by AS (OR 2.02, 95% CI 1.21, 3.38, *p* < 0.05) and SSF (OR 1.17, 95% CI 0.97, 1.41, *p* = 0.56), compared to controls (Fig. 3).

### Dislocations

**Fig. 4:**
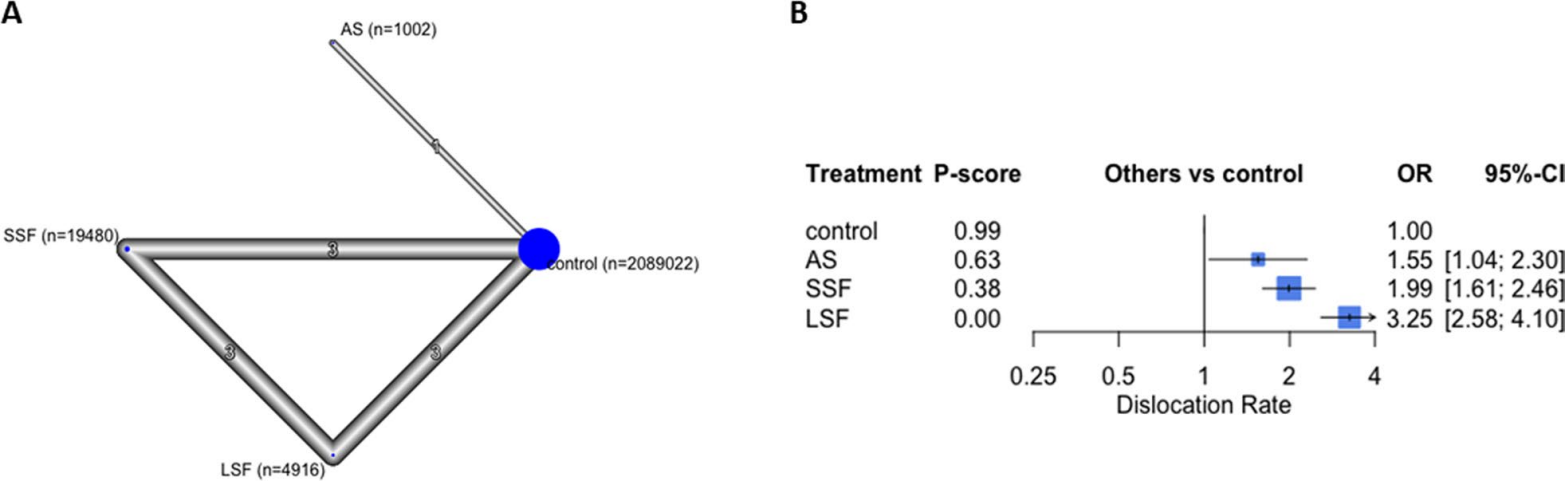
The dislocation rate analysis. In the network plot **a**, each ellipse (node) represents a specific exposure, with its size proportional to the number of cohorts in the node. Connections between nodes represent direct comparative studies between exposures, with the weight of the connecting lines representing the volume of direct comparative evidence. **b** Forest plot depicting the estimated odds ratio (OR) for each exposure pair denoted by the black square, and its associated 95% CI denoted by the width of the line, with a higher OR indicating a higher odd of complications of the group. The higher P-score demonstrates the better exposure. The 95% PI refers to the 95% prediction interval for each point estimate

Eleven studies with 2,114,420 participants reported this outcome [4, 9, 10, 13, 26–32]. LSF reported the highest dislocation rate (OR 3.25; 95% CI 2.58, 4.10, *p* < 0.05), followed by SSF (OR 1.99, 95% CI 1.61, 2.46, *p* < 0.05) and AS (OR 1.55, 95% CI 1.04, 2.30, *p* < 0.05), compared to controls (Fig. 4). The overall structure is shown in Fig. 4.

### Infections

**Fig. 5:**
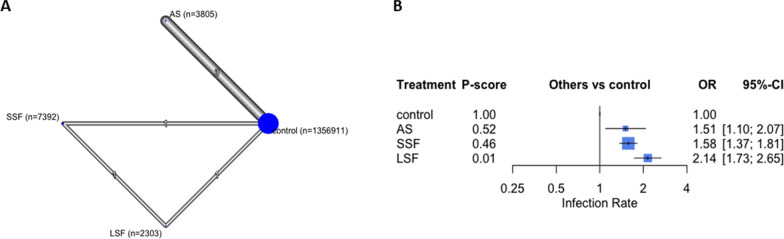
The infection rate analysis. In the network plot **a**, each ellipse (node) represents a specific exposure, with its size proportional to the number of cohorts in the node. Connections between nodes represent direct comparative studies between exposures, with the weight of the connecting lines representing the volume of direct comparative evidence. **b** Forest plot depicting the estimated odds ratio (OR) for each exposure pair denoted by the black square, and its associated 95% CI denoted by the width of the line, with a higher OR indicating a higher odd of complications of the group. The higher P-score demonstrates the better exposure. The 95% PI refers to the 95% prediction interval for each point estimate

Seven studies with 1,370,411 participants reported this outcome [4, 9, 23, 25, 26, 28, 30]. All of the patients were reported as deep infections (periprosthetic joint infection). LSF reported the highest infection rate (OR 2.14; 95% CI 1.73, 2.65, *p* < 0.05), followed by SSF (OR 1.58, 95% CI 1.37, 1.81, *p* < 0.05) and AS (OR 1.51, 95% CI 1.10, 2.07, *p* < 0.05), compared to controls (Fig. 5). The overall structure is shown in Fig. 5.

### Revisions

**Fig. 6:**
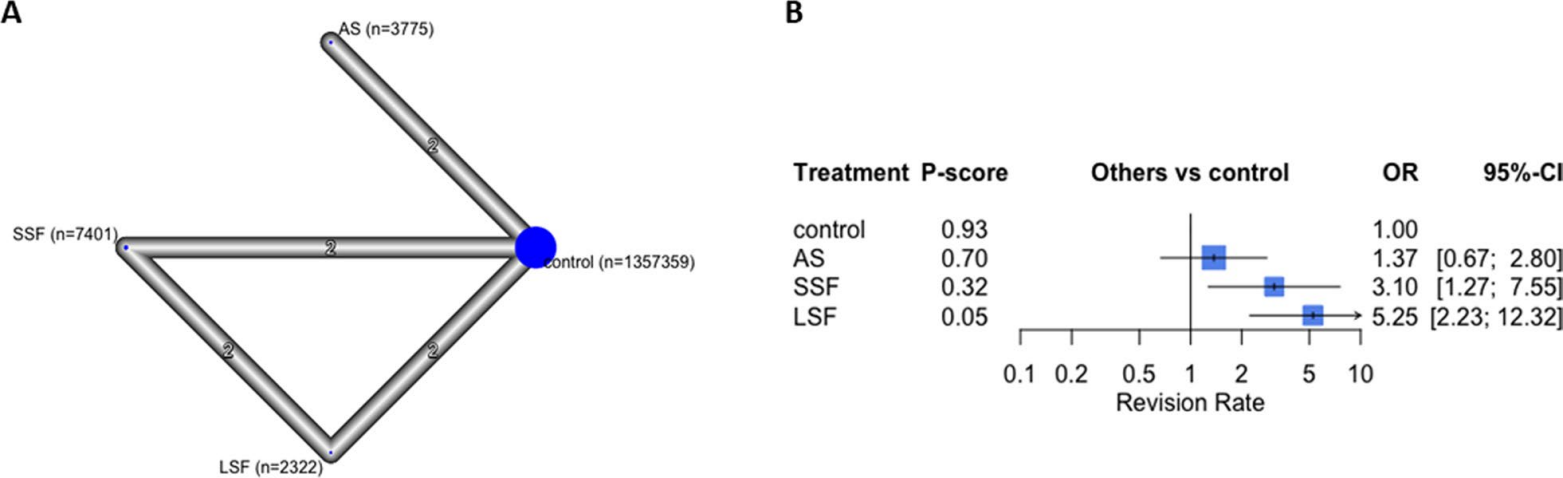
The revision rate analysis. In the network plot **a**, each ellipse (node) represents a specific exposure, with its size proportional to the number of cohorts in the node. Connections between nodes represent direct comparative studies between exposures, with the weight of the connecting lines representing the volume of direct comparative evidence. **b** Forest plot depicting the estimated odds ratio (OR) for each exposure pair denoted by the black square, and its associated 95% CI denoted by the width of the line, with a higher OR indicating a higher odd of complications of the group. The higher P-score demonstrates the better exposure. The 95% PI refers to the 95% prediction interval for each point estimate

Eight studies with 1,370,857 participants reported revision rates [4, 9, 10, 23, 29–32]. Revision rates ranged from 3–32%. Overall, LSF reported the highest revision rate (OR 5.25; 95% CI 2.23, 12.32, *p* < 0.05), followed by SSF (OR 3.10, 95% CI 1.27, 7.55, *p* < 0.05) and AS (OR 1.37, 95% CI 0.67, 2.80, *p* = 0.67), compared to controls (Fig. 3). The overall structure is shown in Fig. 6.

### Post hoc analysis

For potential duplication of database, we identified a potential overlapping of the timing and the database from our mechanical complication outcome. However, after post hoc analysis excluding the other study, the results were similar. After excluding Sing 2016 [9], LSF had the highest mechanical complications (OR 4.81; 95% CI 1.72, 13.47, *p* < 0.05), followed by SSF (OR 3.58; 95% CI 1.22, 10.46, *p* < 0.05), followed by AS (OR 1.61; 95% CI 0.60, 4.34, *p* = 0.67), compared to controls. After excluding Buckland 2017 [10], LSF had the highest mechanical complications (OR 3.04; 95% CI 2.32, 3.98, *p* < 0.05), followed by SSF (OR 1.76; 95% CI 1.35, 2.28, *p* < 0.05), followed by AS (OR 1.45; 95% CI 0.49, 4.32, *p* = 0.65), compared to controls (Additional file 1).

## Discussion

The aim of this systematic review and network meta-analysis was to compare postoperative THA complications among AS patients and non-AS patients with previous SF. Our results demonstrated that among stiff spine patients, SF has increased rates of mechanical complications compared to AS patients. Sub-analysis further identified that patients with long spinal fusion were observed to have the highest rate of both aseptic and septic complications. This evidence not only confirms the stiff spine THA complication relationship but also highlights the importance of identifying and informing patients at high risk of perioperative complications following THA. In a flexible normal spine range of motion, the pelvis tilts posteriorly from standing to sitting, and this in turn enables the hips to flex in order to make the femurs parallel to the ground [33]. Contrastingly, decreased pelvic rollback is observed in a stiff spine range of motion, which increases hip joint flexion in the seated position [34]. This mechanism increases the risk of bony impingement of the anterior acetabular rim and reduces posterior–inferior acetabular coverage of the femoral head in the seated position leading to dislocations [35].

With regard to AS patients, previous literature has reported increased aseptic mechanical THA failure due to destabilizing capsular hypertrophy, fibrosis, abnormal spinopelvic mechanics, and hardware malpositioning [11]. This is likely attributed to a marked positive sagittal balance, hyperextended pelvis, loss of lumbar lordosis, and thoracic kyphotic deformity. The ankylosed restriction of the spine-hip relationship results in hip contractures. Wang et al. reported that 30.7% of AS patients' acetabular cups were placed outside the safe zone [36]. Safe zones function on the basis of a neutral pelvic tilt (PT); however, AS patients have an increased PT. One degree increase in PT correlates with a 0.7 degree of acetabular inclination [33]. Studies have demonstrated that acetabular malpositioning among AS is most likely to be an anteverted and abducted cup, increasing the risk for anterior dislocations [37]. SF patients have a similar issue, with the hip joint as an adjacent motion segment, the long lever arm of a fused spine abnormally loads across the hip, which may lead to early dislocation, loosening, and wear [9]. Our findings demonstrated higher complications among both short and long SF patients when compared to AS and control patients.

The literature remains controversial with regard to the surgical algorithmic approach to minimize spinopelvic alignment-related THA complications among AS and SF patients with concurrent degenerative hip and spine pathologies. The surgical approach may affect the results of THA among previous SF patients. Goyal et al. demonstrated that anterior supine-based approaches including direct anterior and direct lateral for THA had a low risk of instability (< 1%) from a total of 582 previous SF patients [38]. This network meta-analysis demonstrates that the subset cohort of long spinal fusion construct patients present with the greatest risk of postoperative complications following THA. This may be a possibility of higher stiffness among these patients with limited hip motion from long constructs and a fusion to the sacrum or pelvis [30]. Among these patients, additional evaluation is required to identify if spinal deformity correction allows for surgical planning with a constant pelvic incidence and avoids complex THA component positioning [11, 12, 39]. To assist with this decision making, continued efforts are required to integrate advanced comprehensive imaging including three-dimensional imaging for surgical planning and decision making. The functional positioning of the THA implants may reduce the complications [40]. A careful evaluation of the hip spine relationship with supine and standing anteroposterior (AP) pelvis and lateral radiographs with the measure of anterior pelvic plane may be helpful for the classification of the spinal deformity in the surgical planning of THAs [35]. Dual-mobility implant may be suggested in the high dislocation risk patients with a kyphotic lumbar spine and stiff pelvis [41, 42].

Both short and long constructs demonstrated higher risks of aseptic loosening (OR 1.2 and 2.3) and dislocation (OR 2.0 and 3.3) compared to non-SF patients at the final follow-up. This may be attributed to the increased demand across the hip joint with the combination of functional malpositioning [9]. Malpositioning of the acetabular component is a major risk factor of dislocations, loosening, and early wear*.* Klemt et al. retrospectively demonstrated that among 505 revision THA patients with concurrent degenerative lumbar spinal stenosis, patients with prior LSF demonstrated significantly higher rates of dislocation and re-revision rates compared with patients who underwent revision THA followed by LSF [43]. Consideration of options such as variable neck version femoral stems, larger heads, changes in surgical exposure, and dual-mobility articulations in THAs has been previously proposed [44]. This study’s pooled data reported higher dislocation rates in long spinal fusion constructs (7%) compared to AS (5%) and control (3%) patients. Most fusions involve L4-S1, which contribute the greatest spinal effect to both overall spinopelvic motion and the major degrees of freedom for lumbar lordosis.

Our findings support the biomechanical literature that the longer the spinal fusion construct, the higher the dislocation risk when compared to AS and control patients. Sing et al. reported higher THA dislocation rates among patients with longer fusion constructs (more than three levels) compared to patients with short constructs (less than three) in the immediate postoperative period, 6-month and 1-year follow-up time periods [9]. Furthermore, Buckland et al. observed a 3.13 and 1.87 increased risk of THA dislocation among patients with 3- to 7-level fusion and 2-level fusion, respectively [10]. Although our results and the biomechanical literature may be suggesting the potential differences in the complication rates for AS, SSF, LSF patients compared to the non-rigid population due to stiff spines, poorer general conditions may have also contributed to the THA failures.

When evaluating septic complications, AS, LSF, and SSF all reported higher rates compared to controls. Ma et al. concluded that although AS patients require tumor necrosis factor (TNF) inhibitors and high-dose steroids for effective treatment management [45], both medications are known risk factors for THA PJI [46]. Yang et al. reported that among 85,595 Medicare patients who underwent THA prior to SF reported a 2.65 increased risk of infection compared to patients who underwent SF followed by THA [47]. Sing et al. showed that LSF may lead to decreased mobility and physical activity precipitating increased sedentary behaviors, lower postoperative recovery, higher rates of placement to skilled nursing facilities, and subsequent periprosthetic joint infection [9]. These findings may also be attributed to exposure complexity, longer operative time, and bleeding complications among LSF patients. Continued efforts and randomized control trials are required to further investigate the surgical strategy to decrease infection rates of patients with dual hip and spinal degenerative disease.

To our knowledge, this is the first network meta-analysis to measure both direct and indirect comparisons among stiff spine patients undergoing THAs. The weakness of this study is to be noted that most studies included in this network meta-analysis are from USA-based studies. Some European studies have focused on the issue but were mostly review articles, making the difficulty to compare the geographical and cultural differences in this issue [48, 49]. The strength of this study is that we are given a large total number of 3,418,499 stiff spine patients from the existing literature. No articles reported a higher number compared to ours for the comparison of SF and AS [40]. However, some limitations must be addressed as well. First, our patient population was heterogeneous. Although studies collected targeted stiff spine cohorts, some cohorts included avascular necrosis and inflammatory arthritis of the hip in addition to the commonly presented osteoarthritic hip in AS and SF patients. To account for potential confounders including age, sex, race, and additional comorbidities, propensity score matching was implemented in the majority of included cohort and registry studies. Moreover, stiff spines are defined as a less than 10-degree change in sacral slope from standing to sitting reported in the literature [35]. However, we assume AS and SF patients have stiffer spine constructs compared to the general population. Although SF patients are stratified as short or long constructs, it was difficult to stratify the severity of AS into more or less stiff spines. Most of the AS studies included registry data with the extraction and analysis of ICD 9 or 10 codes, which do not clearly define the classification of the stiff spine. Also, the influence of the spinal pathology on the outcomes among the non-AS and non-SF populations remains unclear. Second, owing to the limitations in most national databases, most studies lack radiographic assessment of whether the spine was fused to the sacrum or pelvis, and this may also influence the dislocation rates. Third, most studies did not provide a detailed explanation of the THA implant used or the surgical approach, both of which are factors associated with postoperative outcomes. Finally, none of the studies included were randomized controlled trials, which would be considered as higher evidence for meta-analysis studies. Network meta-analysis should be better done with randomized controlled trials, with a result of decreasing the potential violation of transitivity. Furthermore, we assumed AS as a type of exposure to the patients similar to interventions such as SF involving the spine and hip. This may have resulted in a potential bias toward the results of this study. However, our study findings are important for clinicians such as hip and spine surgeons for the awareness of spinopelvic mobility and the effects of spinal pathology or fusion on the limiting compensatory motion mechanism contributing to complications. Further development of a functional assessment tool to validate the treatment algorithms may lead to improved evidence-based practices for hip and spine surgeons. A thorough discussion with the patients regarding expectations, timing of surgery, preoperative planning including radiographic assessments, explanation of the increased risk of postoperative complications, and management strategies should occur preoperatively [50]. Further research should be done to better understand the spinopelvic relationship among the stiff spine population and with more randomized controlled study designs with this issue. Technology improvement including intraoperative imaging and the robotic or computer-assisted surgery may improve the accuracy of acetabular component placement [50–52]. Operators should be cautious when performing THAs on long spinal fusion patients.

## Conclusion

In summary, this network meta-analysis demonstrated that, among the stiff spine population, long SF constructs have a higher risk of complications compared to short SF constructs, AS patients, and the general population undergoing THA. Surgeons performing THAs should be aware of this subset of patients and understand the spinopelvic relationship of stiff spines.

## Supplementary Information

**Additional file 1: Figure S1**. Publication bias. **Table S1**. Electronic search strategy. ** Table S2**. Risk of bias assessment according to Newcastle-Ottawa Quality Assessment Scale for cohort studies.**Table S3**. Assessment of the quality of included studies by GRADE. **Table S4**. Pairwise meta-analysis of odds ratio (95% CI).

## Data Availability

All data generated or analyzed during this study are included in this published article and its Additional files.

## Abbreviations

AS: Ankylosing spondylitis
SF: Spinal fusion
SSF: Short spinal fusion
LSF: Long spinal fusion
HO: Heterotopic ossification
MeSH: Medical Subject Headings
THA: Total hip arthroplasty
PT: Pelvic tilt
AP: Anteroposterior
CI: Confidence interval
TNF: Tumor necrosis factor
PICO: Patient–Intervention–Comparison–Outcome
PRISMA: Preferred Reporting Items for Systematic Reviews and Meta-Analyses
OR: Odds ratio
