# Supplementary material for: Total hip arthroplasty has higher complication rates in stiff spine patients: a systematic review and network meta-analysis
Source: J Orthop Surg Res. 2022 Jul 16;17:353. doi: 10.1186/s13018-022-03237-8 (PMC9288065; doi:10.1186/s13018-022-03237-8)
Supplement: Supplementary file 1 — Additional file 1: Figure S1. Publication bias. Table S1. Electronic search strategy. Table S2. Risk of bias assessment according to Newcastle-Ottawa Quality Assessment Scale for cohort studies.Table S3. Assessment of the quality of included studies by GRADE. Table S4. Pairwise meta-analysis of odds ratio (95% CI). [file 13018_2022_3237_MOESM1_ESM.docx]

**SUPPLEMENTARY CONTENT**

**The following material accompanies the article “Total Hip Arthroplasty in Stiff Spines have Higher Complication Rates: A Systematic Review and Network Meta-Analysis”**

**Table of Content**

**Search strategy and exclusion of studies with reasons p. 3-4**

**Risk of bias assessment of Cohort Studies p. 5-6**

**Assessment of quality of included studies p.7**

**Pairwise meta-analysis p.8**

**Publication Bias assessment p.9**

**Subgroup analysis p.10-12**

**PRISMA checklist p.13**

**Supplementary Table 1: Electronic search strategy**

| **MEDLINE** |
| --- |
| 1. "arthroplasty, replacement, hip"[MeSH Terms] OR ("arthroplasty"[All Fields] AND "replacement"[All Fields] AND "hip"[All Fields]) OR "hip replacement arthroplasty"[All Fields] OR ("total"[All Fields] AND "hip"[All Fields] AND "arthroplasty"[All Fields]) OR "total hip arthroplasty"[All Fields] 2. "arthroplasty, replacement, hip"[MeSH Terms] OR ("arthroplasty"[All Fields] AND "replacement"[All Fields] AND "hip"[All Fields]) OR "hip replacement arthroplasty"[All Fields] OR ("total"[All Fields] AND "hip"[All Fields] AND "replacement"[All Fields]) OR "total hip replacement"[All Fields] 3. "spondylitis, ankylosing"[MeSH Terms] OR ("spondylitis"[All Fields] AND "ankylosing"[All Fields]) OR "ankylosing spondylitis"[All Fields] OR ("ankylosing"[All Fields] AND "spondylitis"[All Fields]) OR ("spinal fusion"[All Fields] OR " lumbar fusion "[All Fields] OR " thoracolumbar fusion"[All Fields] OR "thoracic fusion"[All Fields]) 4. "dislocate"[All Fields] OR "dislocates"[All Fields] OR "dislocating"[All Fields] OR "dislocator"[All Fields] OR "dislocators"[All Fields] OR "joint dislocations"[MeSH Terms] OR ("joint"[All Fields] AND "dislocations"[All Fields]) OR "joint dislocations"[All Fields] OR "dislocated"[All Fields] OR "dislocation"[All Fields] OR "dislocations"[All Fields] 5. "reoperate"[All Fields] OR "reoperated"[All Fields] OR "reoperating"[All Fields] OR "reoperation"[MeSH Terms] OR "reoperation"[All Fields] OR "reoperations"[All Fields] OR "reoperative"[All Fields] OR "second look surgery"[MeSH Terms] OR ("second look"[All Fields] AND "surgery"[All Fields]) OR "second look surgery"[All Fields] 6. "revise"[All Fields] OR "revised"[All Fields] OR "revisers"[All Fields] OR "revises"[All Fields] OR "revising"[All Fields] OR "revision"[All Fields] OR "revisions"[All Fields] 7. "loosen"[All Fields] OR "loosened"[All Fields] OR "loosening"[All Fields] OR "loosenings"[All Fields] OR "loosens"[All Fields] 8. "infect"[All Fields] OR "infectability"[All Fields] OR "infectable"[All Fields] OR "infectant"[All Fields] OR "infectants"[All Fields] OR "infected"[All Fields] OR "infecteds"[All Fields] OR "infectibility"[All Fields] OR "infectible"[All Fields] OR "infecting"[All Fields] OR "infection s"[All Fields] OR "infections"[MeSH Terms] OR "infections"[All Fields] OR "infection"[All Fields] OR "infective"[All Fields] OR "infectiveness"[All Fields] OR "infectives"[All Fields] OR "infectivities"[All Fields] OR "infects"[All Fields] OR "pathogenicity"[MeSH Subheading] OR "pathogenicity"[All Fields] OR "infectivity"[All Fields] 9. "arthroplasty, replacement, hip"[MeSH Terms] OR ("arthroplasty"[All Fields] AND "replacement"[All Fields] AND "hip"[All Fields]) OR "hip replacement arthroplasty"[All Fields] OR ("total"[All Fields] AND "hip"[All Fields] AND "arthroplasty"[All Fields]) OR "total hip arthroplasty"[All Fields] OR ("arthroplasty, replacement, hip"[MeSH Terms] OR ("arthroplasty"[All Fields] AND "replacement"[All Fields] AND "hip"[All Fields]) OR "hip replacement arthroplasty"[All Fields] OR ("total"[All Fields] AND "hip"[All Fields] AND "replacement"[All Fields]) OR "total hip replacement"[All Fields]) 10. #1 OR #2 11. #4 OR #5 OR #6 OR #7 OR #8 12. #9 AND #3 AND #10 |
| **EMBASE** |
| 1. ('total hip replacement'/exp OR 'hip total arthroplasty' OR 'hip total joint replacement' OR 'hip total replacement' OR 'hip total replacement arthroplasty' OR 'total hip replacement' OR 'total replacement hip arthroplasty' OR 'total hip arthroplasty') 2. ('ankylosing spondylitis'/exp OR 'bechterew disease' OR 'ankylating spondylitis' OR 'ankylopoietic spondylarthritis' OR 'ankylopoietic spondylitis' OR 'ankylosing spine' OR 'ankylosing spondilitis' OR 'ankylosing spondylarthritis' OR 'ankylosing spondylarthrosis' OR 'ankylosing spondylitis' OR 'ankylosis spondylitis' OR 'ankylotic spondylitis' OR 'bekhterev disease' OR 'morbus bechterew' OR 'spinal ankylosis' OR 'spine ankylosis' OR 'spondylarthritis ankylopoietica' OR 'spondylarthritis ankylosans' OR 'spondylarthrosis ankylopoietica' OR 'spondylitis ankylopoetica' OR 'spondylitis ankylopoietica' OR 'spondylitis, ankylosing' OR 'spondyloarthritis ankylopoietica' OR 'vertebral ankylosis') 3. ('dislocation'/exp OR 'dislocation' OR 'dislocations' OR 'luxation' OR 'semitunar bone dislocation' OR 'reoperation'/exp OR 'reoperation' OR 'revision'/exp OR loosening OR 'infection'/exp OR 'accidental infection' OR 'acute infection' OR 'autoinfection' OR 'bacterial infections and mycoses' OR 'bacteroid infection' OR 'chain of infection' OR 'chronic infection' OR 'dormant infection' OR 'focal infection' OR 'inapparent infection' OR 'infection' OR 'infection mechanism' OR 'infection route' OR 'infection, focal' OR 'infections' OR 'infectious disease' OR 'infectivity' OR 'latent infection' OR 'route of infection' OR 'silent infection' OR 'simultaneous infection') 4. ('spinal fusion'/exp OR' lumbar fusion'/exp OR 'thoracolumbar fusion'/exp OR 'thoracic fusion'/exp) 5. 1 and (2 OR 4) and 3 |
| **Cochrane CENTRAL** |
| 1. total hip arthroplasty 2. MeSH descriptor: [Arthroplasty, Replacement, Hip] explode all trees 3. total hip replacement 4. #1 OR #2 OR #3 5. Ankylosing Spondylitis 6. MeSH descriptor: [Spondylitis, Ankylosing] explode all trees 7. #5 OR #6 8. dislocation 9. MeSH descriptor: [Hip Dislocation] explode all trees 10. #8 OR #9 11. loosening 12. prosthetic joint infection 13. hip revision 14. Spinal Fusion 15. #10 OR #11 OR #12 OR #13 16. #4 AND (#7 OR #14) AND #15 |
| **Web of Science** |
| (TS= (infection OR loosening OR dislocation OR revision AND TS=( Ankylosing Spondylitis OR Spinal Fusion) AND TS=( total hip replacement) |
| **Scopus** |
| ( ( TITLE-ABS-KEY ( infection ) OR TITLE-ABS-KEY ( loosening ) OR TITLE-ABS-KEY ( dislocation ) OR TITLE-ABS-KEY ( revision ) ) ) AND ( TITLE-ABS-KEY ( "Ankylosing Spondylitis" ) OR TITLE-ABS-KEY ( "Spinal Fusion" ) ) AND ( ( TITLE-ABS-KEY ( "total hip replacement" ) OR TITLE-ABS-KEY ( "total hip arthroplasty" ) ) ) AND ( LIMIT-TO ( LANGUAGE , "English" ) ) |

**Supplemental Table 2**. Risk of bias assessment according to Newcastle-Ottawa Quality Assessment Scale for cohort studies

| **Study** | **Quality Assessment Criteria** | | | | | | | | |
| --- | --- | --- | --- | --- | --- | --- | --- | --- | --- |
|  | **Selection** | | | | **Comparability** | **Outcome** | | | **Total Score** |
|  | **Representativeness of Exposed Cohort** | **Selection of non Exposed Cohort** | **Ascertainment of Exposure** | **Outcome of Interest** | **Based on Analysis** | **Assessment of Outcome** | **Follow up length** | **Adequacy of follow up of cohorts** |  |
| Ward 2019 | * | * |  | * |  | * | * | * | 4 |
| Banos 2004 | * | * |  | * |  | * | * | * | 6 |
| Blizzard 2017 | * | * |  | * | * | * | * | * | 7 |
| Lee 2017 | * |  |  | * | * | * | * | * | 8 |
| Katakam 2020 | * |  | * | * |  | * | * | * | 8 |
| Barry 2017 | * |  | * | * | * | * |  |  | 5 |
| Bedard 2016 | * |  | * | * |  | * | * | * | 8 |
| Buckland 2017 | * | * |  | * | * | * | * | * | 7 |
| Loh 2017 | * |  | * | * | * | * | * | * | 7 |
| York 2018 | * |  | * | * |  | * | * | * | 6 |
| Salib 2019 | * | * | * | * | * | * | * | * | 8 |
| Sing 2016 | * | * |  | * |  | * | * | * | 6 |
| Perfetti 2017 | * | * |  | * | * | * | * | * | 7 |
| Malkani 2018 | * | * |  | * | * | * | * | * | 7 |

**Selection:
a) Representativeness of Exposed Cohort:** Score given if truly representative of the community
**b) Selection of non Exposed Cohort:** Score given if drawn from the same community
**c) Ascertainment of Exposure:** Score given if secure record
**d) Outcome of Interest:** Score given if outcome of interest was not present at start of study
2.**Comparability:** Score given if study controls for important confounding factors including age and sex
3.**Outcome:**
**a) Assessment of Outcome:** Score given if record linkage is correct **b) Follow up length:** Score given if follow-up long enough for outcomes to occur **c) Adequacy of follow up of cohort:** Score given if complete follow up described in the study

By identifying outcome from ICD-9 or CPT code, Ward 2019, Blizzard 2017, Buckland 2017, Perfetti 2017, and Sing 2016 lead to concerns of lacking ascertainment of exposure. There was no description of whether structured interview where blind to case/control status in Katakam 2020, York 2018, Barry 2017, Lee 2017, Loh 2017, and Bedard 2016, posting potential risk of bias. No matching details were recorded in Ward 2019, Banos 2004, Katakam 2020, Bedard 2016, York 2018, or Sing 2016. However, most characteristic item between the 2 groups did not reach statistical significance. Ward 2019, Blizzard 2017, Buckland 2017, Perfetti 2017, and Sing 2016 identified patient group by using both The International Classification of Diseases, Ninth Revision, (ICD-9) diagnosis code and procedure code, while Malkani 2018 used Current Procedural Terminology (CPT) code only, leading to concerns of lacking independent validation among studies. Lee 2017 did not specify the definition and inclusion criteria for both AS and control group. In Banos 2004, control group was identified as patients with rheumatoid arthritis (RA), juvenile chronic arthritis (JCA), and osteoarthritis (OA), which may post concerns for representativeness of the general population.


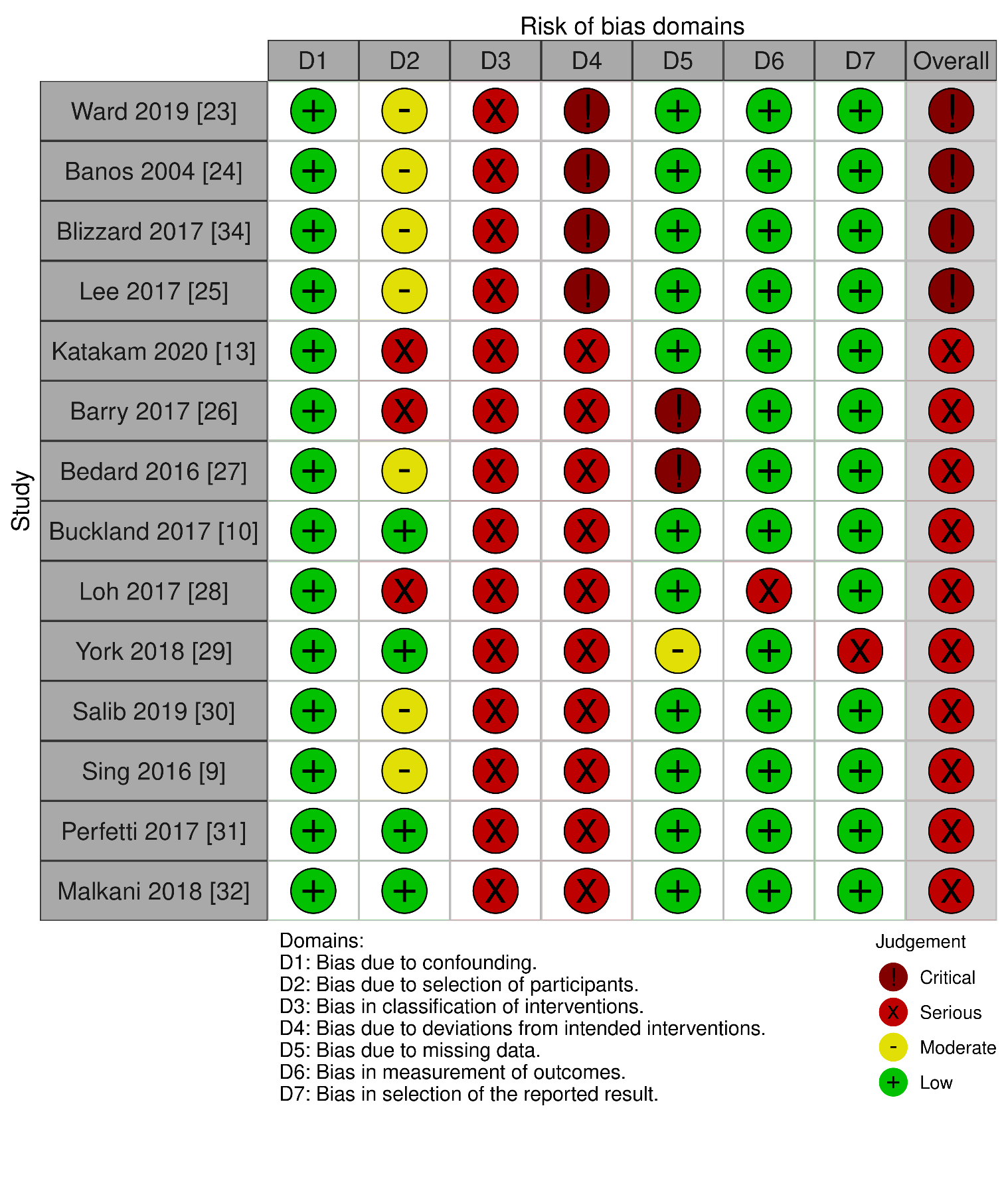


**Supplemental Table 3**. Assessment of the quality of included studies by GRADE

| **Outcome** | **Quality Assessment Criteria** | | | | | | | |
| --- | --- | --- | --- | --- | --- | --- | --- | --- |
|  | **Number of Studies** | **Number of Patients** | **Inconsistency** | **Indirectness** | **Imprecision** | **Other consideration (Biases)** | **Relative Effect (95% CI)** | **Confidence to Effect Estimates (GRADE)** |
| Mechanical Complications | 14 | 2,348,505 | Serious | Not serious | Not serious | Not serious | SF  2.33(1.86,2.92)  AS  1.18(0.87,1.61) | Moderate |
| Aseptic Loosening | 6 | 1,260,419 | Serious | Not serious | Not serious | Not serious | LSF  2.33(1.83,2.95)  AS  2.02(1.21,3.38)  SSF  1.17(0.97,1.41) | Moderate |
| Dislocations | 10 | 2,114,420 | Serious | Not serious | Not serious | Not serious | LSF  3.25(2.58,4.10)  SSF  1.99(1.61,2.46)  AS  1.55(1.04,2.30) | Moderate |
| Infections | 6 | 1,370,411 | Serious | Not serious | Not serious | Not serious | LSF  2.14(1.73,2.65)  SSF  1.58(1.37,1.81)  AS  1.51(1.10,2.07) | Moderate |
| Revisions | 8 | 1,370,857 | Serious | Not serious | Not serious | Not serious | LSF  5.25(2.23,12.32)  SSF  3.10(1.27,7.55)  AS  1.37(0.67,2.80) | Moderate |

**Supplementary Table 4: Pairwise meta-analysis of odds ratio (95% CI)**

| **Comparison** | | **Pairwise meta-analysis** | **No of participants** | **No. of studies** | **Heterogeneity I^2^** |
| --- | --- | --- | --- | --- | --- |
| Aseptic Loosening | | | | | |
| AS | Control | 2.02 (1.21 to 3.38) | 1049 | 3 | 0% |
| SSF |  | 1.17 (0.97 to 1.41) | 7392 | 1 | NA |
| LSF |  | **2.33 (1.83 to 2.95)** | 2303 | 2 | NA |
| SF |  | **1.74 (1.18 to 2.56)** | 9827 | 3 | 0% |
| SSF | LSF | **0.50 (0.37 to 0.68)** | 9695 | 1 | NA |
| Dislocations | | | | | |
| AS | Control | **1.55 (1.17 to 2.04)** | 1002 | 1 | NA |
| SSF |  | 2.87 (0.27 to 30.52) | 19480 | 3 | 70% |
| LSF |  | 4.05 (0.76 to 21.52) | 4916 | 2 | 0% |
| SF |  | **2.72 (1.39 to 5.33)** | 27396 | 9 | 74% |
| SSF | LSF | 0.63 (0.39 to 1.00) | 24396 | 3 | 41% |
| Infections | | | | | |
| AS | Control | 1.49 (0.73 to 3.03) | 3805 | 3 | 0% |
| SSF |  | **1.58 (1.37 to 1.81)** | 7392 | 1 | NA |
| LSF |  | **2.14 (1.73 to 2.65)** | 2303 | 1 | NA |
| SF |  | 3.21 (0.19 to 54.52) | 9827 | 4 | 40% |
| SSF | LSF | **0.73 (0.57 to 0.95)** | 9695 | 1 | NA |
| Revisions | | | | | |
| AS | Control | 1.37 (0.20 to 9.26) | 3775 | 2 | 73% |
| SSF |  | 5.68 (0.00 to 2582132) | 7401 | 2 | 89% |
| LSF |  | 9.41 (0.00 to 9939336) | 2322 | 2 | 95% |
| SF |  | **3.79 (1.12 to 12.81)** | 27231 | 6 | 97% |
| SSF | LSF | **0.70 (0.60 to 0.81)** | 9723 | 2 | 0% |

**Supplementary Figure 1: Publication bias**

| **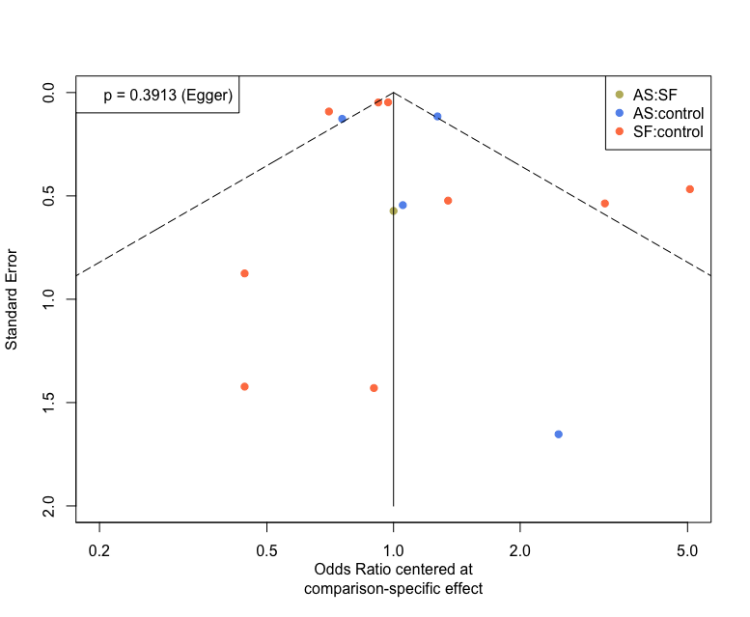** | **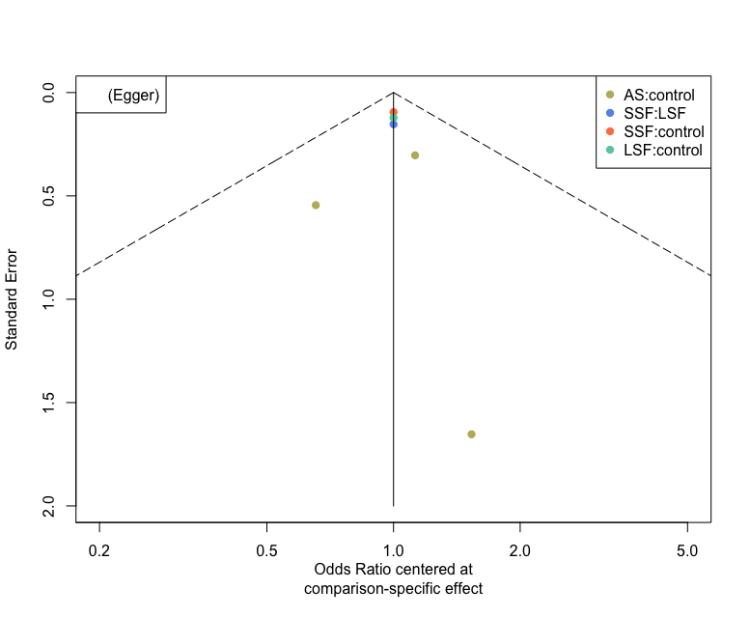** | **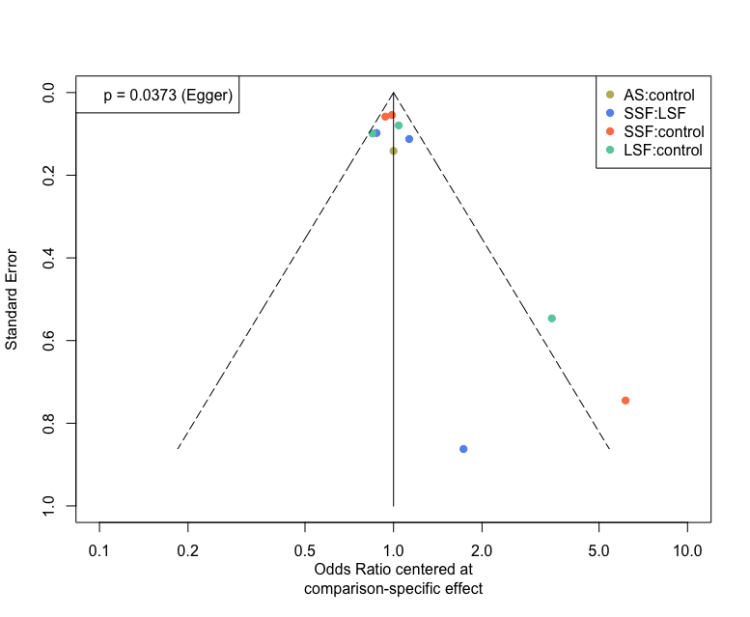** |
| --- | --- | --- |
| Mechanical Complications | Aseptic Loosening | Dislocation |
| **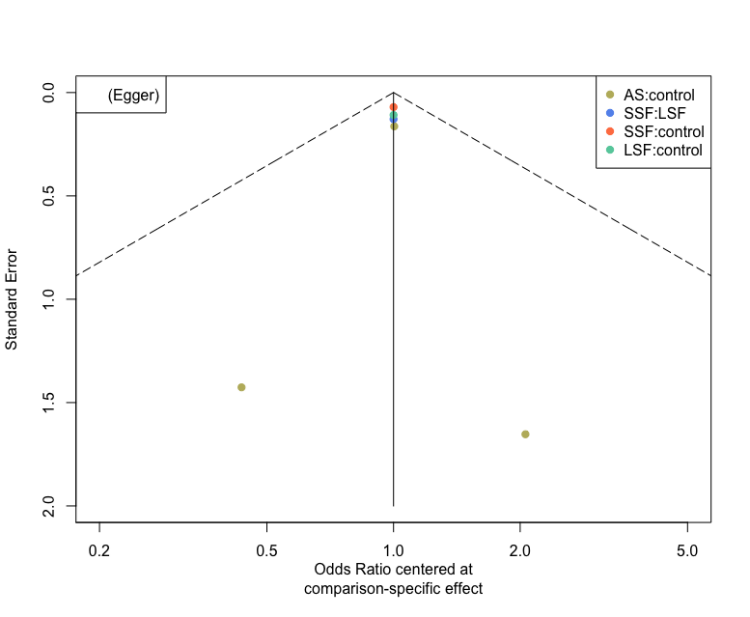** | 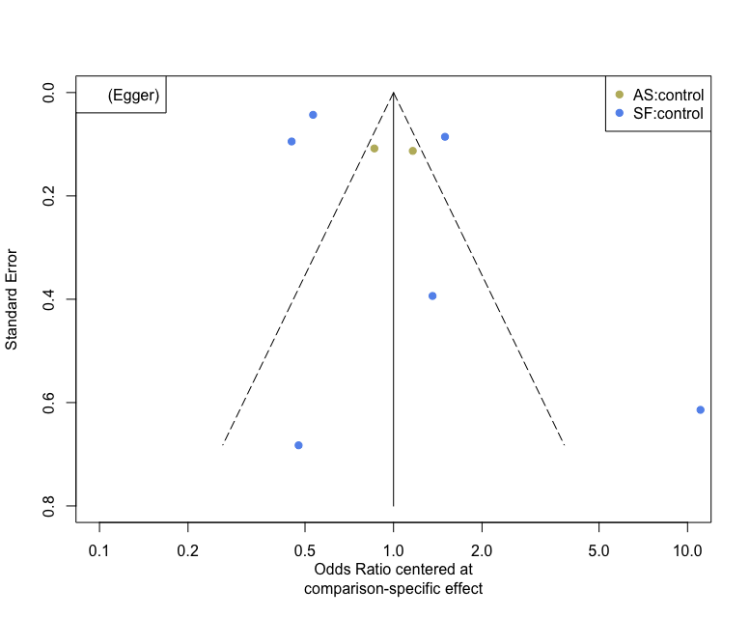 |  |
| Infection | Revision |  |

**Supplementary Table 5: Potential overlap of database**

| **Study** | **Group** | **Location** | **Database** |
| --- | --- | --- | --- |
| Ward 2019 [20] | AS | USA | 1999 to 2013 of US Medicare |
| Banos 2004 [21] | AS | Argentina |  |
| Blizzard 2017 [31] | AS | USA | 2005 to 2012 of US Medicare |
| Lee 2017 [22] | AS | Korea |  |
| Katakam 2020 [13] | AS,SF | USA | 2000 to 2017 of Institutional Data (MGH) |
| Barry 2017 [23] | SF | USA | 2012 to 2015 of Institutional Data (UCSF) |
| Bedard 2016 [24] | SF | USA | 2004 to 2014 of Institutional Data (Iowa Hospital) |
| Buckland 2017 [10] | SSF,LSF | USA | 2005 to 2012 of US Medicare |
| Loh 2017 [25] | SF | Singapore |  |
| York 2018 [26] | SSF,LSF | USA | 2010 to 2014 of Institutional Data (UC Denver) |
| Salib 2019 [27] | SF | USA | 1998 to 2015 of Institutional Data (Mayo Clinic) |
| Sing 2016 [9] | SSF,LSF | USA | 2005 to 2012 of PearlDiver (US Medicare) |
| Perfetti 2017 [28] | SF | USA | 2005 to 2012 of SPARCS New York |
| Malkani 2018 [29] | SF | USA | 2002 to 2014 of US Medicare |

**Supplementary Figure 2: Subgroup analysis of mechanical complications excluding Sing 2016 due to potential overlap of database**

**
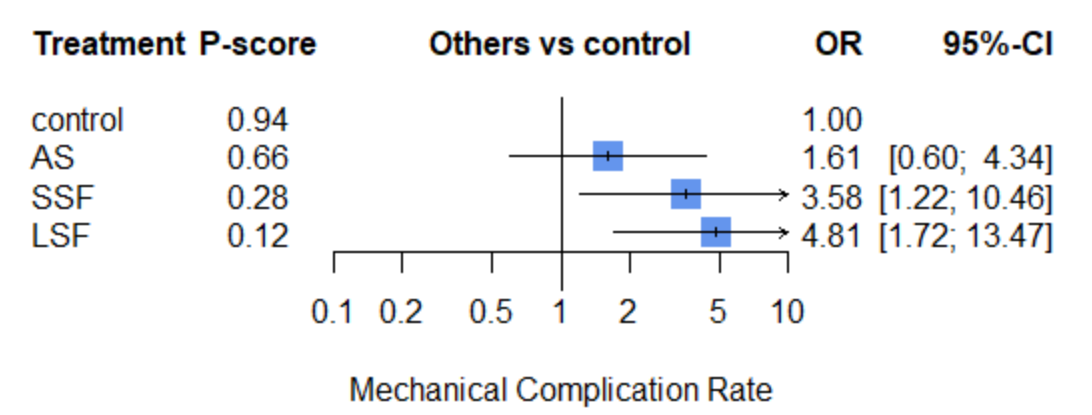
**

**Supplementary Figure 3: Subgroup analysis of mechanical complications excluding Buckland 2017 due to potential overlap of database**


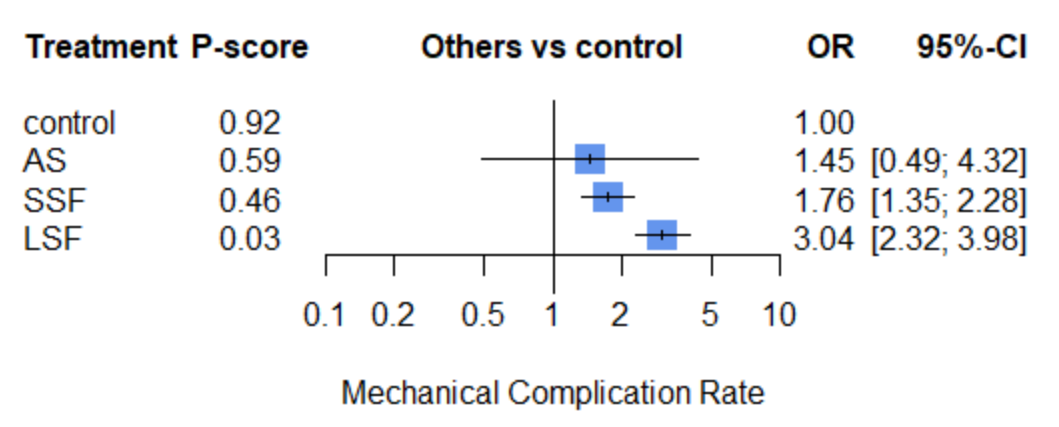


**PRISMA checklist**

| **Section and Topic** | **Item #** | **Checklist item** | **Location where item is reported** |
| --- | --- | --- | --- |
| **TITLE** | | |  |
| Title | 1 | Identify the report as a systematic review. | P1 |
| **ABSTRACT** | | |  |
| Abstract | 2 | See the PRISMA 2020 for Abstracts checklist. | P2 |
| **INTRODUCTION** | | |  |
| Rationale | 3 | Describe the rationale for the review in the context of existing knowledge. | P3 |
| Objectives | 4 | Provide an explicit statement of the objective(s) or question(s) the review addresses. | P3 |
| **METHODS** | | |  |
| Eligibility criteria | 5 | Specify the inclusion and exclusion criteria for the review and how studies were grouped for the syntheses. | P4- *Research Protocol and Search Question* |
| Information sources | 6 | Specify all databases, registers, websites, organisations, reference lists and other sources searched or consulted to identify studies. Specify the date when each source was last searched or consulted. | P5- *Search Strategy and Study Selection* |
| Search strategy | 7 | Present the full search strategies for all databases, registers and websites, including any filters and limits used. | P5- *Search Strategy and Study Selection* |
| Selection process | 8 | Specify the methods used to decide whether a study met the inclusion criteria of the review, including how many reviewers screened each record and each report retrieved, whether they worked independently, and if applicable, details of automation tools used in the process. | P5- *Search Strategy and Study Selection* |
| Data collection process | 9 | Specify the methods used to collect data from reports, including how many reviewers collected data from each report, whether they worked independently, any processes for obtaining or confirming data from study investigators, and if applicable, details of automation tools used in the process. | P5- *Data Collection and Quality Assessment* |
| Data items | 10a | List and define all outcomes for which data were sought. Specify whether all results that were compatible with each outcome domain in each study were sought (e.g. for all measures, time points, analyses), and if not, the methods used to decide which results to collect. | P5- *Data Collection and Quality Assessment* |
|  | 10b | List and define all other variables for which data were sought (e.g. participant and intervention characteristics, funding sources). Describe any assumptions made about any missing or unclear information. | P5- *Data Collection and Quality Assessment* |
| Study risk of bias assessment | 11 | Specify the methods used to assess risk of bias in the included studies, including details of the tool(s) used, how many reviewers assessed each study and whether they worked independently, and if applicable, details of automation tools used in the process. | P5- *Data Collection and Quality Assessment* |
| Effect measures | 12 | Specify for each outcome the effect measure(s) (e.g. risk ratio, mean difference) used in the synthesis or presentation of results. | P6- *Statistical Analysis and Quantitative Data Synthesis* |
| Synthesis methods | 13a | Describe the processes used to decide which studies were eligible for each synthesis (e.g. tabulating the study intervention characteristics and comparing against the planned groups for each synthesis (item #5)). | P6- *Statistical Analysis and Quantitative Data Synthesis* |
|  | 13b | Describe any methods required to prepare the data for presentation or synthesis, such as handling of missing summary statistics, or data conversions. | P6- *Statistical Analysis and Quantitative Data Synthesis* |
|  | 13c | Describe any methods used to tabulate or visually display results of individual studies and syntheses. | P6- *Statistical Analysis and Quantitative Data Synthesis* |
|  | 13d | Describe any methods used to synthesize results and provide a rationale for the choice(s). If meta-analysis was performed, describe the model(s), method(s) to identify the presence and extent of statistical heterogeneity, and software package(s) used. | P6- *Statistical Analysis and Quantitative Data Synthesis* |
|  | 13e | Describe any methods used to explore possible causes of heterogeneity among study results (e.g. subgroup analysis, meta-regression). | P6- *Statistical Analysis and Quantitative Data Synthesis* |
|  | 13f | Describe any sensitivity analyses conducted to assess robustness of the synthesized results. | P6- *Statistical Analysis and Quantitative Data Synthesis* |
| Reporting bias assessment | 14 | Describe any methods used to assess risk of bias due to missing results in a synthesis (arising from reporting biases). | P5- *Data Collection and Quality Assessment* |
| Certainty assessment | 15 | Describe any methods used to assess certainty (or confidence) in the body of evidence for an outcome. | P5- *Data Collection and Quality Assessment* |
| **RESULTS** | | |  |
| Study selection | 16a | Describe the results of the search and selection process, from the number of records identified in the search to the number of studies included in the review, ideally using a flow diagram. | P7- *Literature Search and Selection Process* |
|  | 16b | Cite studies that might appear to meet the inclusion criteria, but which were excluded, and explain why they were excluded. | P7- *Literature Search and Selection Process* |
| Study characteristics | 17 | Cite each included study and present its characteristics. | P7- *Study Characteristics, Cohort Description and Treatment Definition* |
| Risk of bias in studies | 18 | Present assessments of risk of bias for each included study. | P8- *Methodological Quality and Assessment of Risk of Bias* |
| Results of individual studies | 19 | For all outcomes, present, for each study: (a) summary statistics for each group (where appropriate) and (b) an effect estimate and its precision (e.g. confidence/credible interval), ideally using structured tables or plots. | P7 |
| Results of syntheses | 20a | For each synthesis, briefly summarise the characteristics and risk of bias among contributing studies. | P7 |
|  | 20b | Present results of all statistical syntheses conducted. If meta-analysis was done, present for each the summary estimate and its precision (e.g. confidence/credible interval) and measures of statistical heterogeneity. If comparing groups, describe the direction of the effect. | P7 |
|  | 20c | Present results of all investigations of possible causes of heterogeneity among study results. | P7 |
|  | 20d | Present results of all sensitivity analyses conducted to assess the robustness of the synthesized results. | P7 |
| Reporting biases | 21 | Present assessments of risk of bias due to missing results (arising from reporting biases) for each synthesis assessed. | P7 |
| Certainty of evidence | 22 | Present assessments of certainty (or confidence) in the body of evidence for each outcome assessed. | P7 |
| **DISCUSSION** | | |  |
| Discussion | 23a | Provide a general interpretation of the results in the context of other evidence. | P8 |
|  | 23b | Discuss any limitations of the evidence included in the review. | P8 |
|  | 23c | Discuss any limitations of the review processes used. | P8 |
|  | 23d | Discuss implications of the results for practice, policy, and future research. | P8 |
| **OTHER INFORMATION** | | |  |
| Registration and protocol | 24a | Provide registration information for the review, including register name and registration number, or state that the review was not registered. | P4- *Research Protocol and Search Question* |
|  | 24b | Indicate where the review protocol can be accessed, or state that a protocol was not prepared. | P4- *Research Protocol and Search Question* |
|  | 24c | Describe and explain any amendments to information provided at registration or in the protocol. | P4- *Research Protocol and Search Question* |
| Support | 25 | Describe sources of financial or non-financial support for the review, and the role of the funders or sponsors in the review. | P1 |
| Competing interests | 26 | Declare any competing interests of review authors. | P1 |
| Availability of data, code and other materials | 27 | Report which of the following are publicly available and where they can be found: template data collection forms; data extracted from included studies; data used for all analyses; analytic code; any other materials used in the review. | P1 |

*From:*  Page MJ, McKenzie JE, Bossuyt PM, Boutron I, Hoffmann TC, Mulrow CD, et al. The PRISMA 2020 statement: an updated guideline for reporting systematic reviews. BMJ 2021;372:n71. doi: 10.1136/bmj.n71

For more information, visit: <http://www.prisma-statement.org/>
